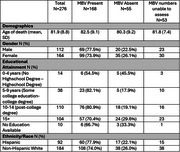# Associations of mineralized blood vessels in an autopsy cohort of persons with Alzheimer Disease who identify as Hispanic and Non‐Hispanic White

**DOI:** 10.1002/alz70855_106000

**Published:** 2025-12-24

**Authors:** Shyam Tamizharasu, David Garcia, Naomi Saito, Melanie N. Luu, Laurel Beckett, Lawrence S. Honig, Charles Decarli, Robert A. Rissman, Andrew F. Teich, Dan M. Mungas, Lee‐Way Jin, Brittany N Dugger, Hsin‐Pei Wang

**Affiliations:** ^1^ University of California, Davis, Sacramento, CA, USA; ^2^ University of California, Davis, Davis, CA, USA; ^3^ Taub Institute for Research on Alzheimer's Disease and the Aging Brain, Columbia University, New York, NY, USA; ^4^ University of California, San Diego, La Jolla, CA, USA; ^5^ Taub Institute for Research on Alzheimer's Disease and the Aging Brain, New York, NY, USA; ^6^ Indiana University, Indianapolis, IN, USA; ^7^ University of California, Davis, School of Medicine, Sacramento, CA, USA

## Abstract

**Background:**

Mineralized blood vessels (MBV) may result in the stiffening of blood vessels and are common in various neurodegenerative disorders, including Alzheimer disease (AD). Despite their frequency, there is limited research on MBV in postmortem neuropathological studies particularly in persons other than non‐Hispanic Whites.

**Method:**

To fill this gap, we examined a cohort of Hispanic decedents HD (*n* =  92) and non‐Hispanic White decedents NHWD (*n* =  184) with pathologically confirmed AD from three Alzheimer's Disease Research Centers: Columbia University, University of California San Diego, and University of California Davis. A neuropathology expert denoted the presence/absence of MBV in hematoxylin and eosin‐stained formalin fixed paraffin embedded sections from three anatomic brain regions: posterior hippocampus, putamen, and globus pallidus.

**Result:**

The highest prevalence of MBV was in the globus pallidus (59.0%), followed by posterior hippocampus (28.6%), and then putamen (14.8%). MBV burden differed among brain regions, with a higher presence of MBV in the: globus pallidus compared to putamen (*p* =  <.001), posterior hippocampus compared to putamen (*p* =  <.001), and globus pallidus compared to posterior hippocampus (*p* =  <.001), by McNemar's test. There were no statistically significant differences with age at death or sex (all *p*‐values >0.1) based on overall presence/absence of MBV. Examining overall MBV presence, HD had a higher frequency (77.9%) than NHWD (74.0%) but was not statistically significant per the Pearson's Chi‐squared test (*p* = .63). There were not significant differences in MBV presence in different brain regions between HD and NHWD, using Pearson's Chi‐squared test.

**Conclusion:**

This study highlights the region‐specific manifestation of MBV, describing an underexplored vascular neuropathology in AD postmortem research. Future work is necessary to understand the role of MBV in the heterogeneous neuropathological landscape of AD and related dementias.